# A minor role of asparaginase in predisposing to cerebral venous thromboses in adult acute lymphoblastic leukemia patients

**DOI:** 10.1002/cam4.1094

**Published:** 2017-05-15

**Authors:** Saara Roininen, Outi Laine, Marjut Kauppila, Marko Vesanen, Maria Rämet, Marjatta Sinisalo, Esa Jantunen, Marjaana Säily, Riikka Räty, Erkki Elonen, Ulla Wartiovaara‐Kautto

**Affiliations:** ^1^ Comprehensive Cancer CenterDepartment of HematologyHelsinki University HospitalHelsinkiFinland; ^2^ University of HelsinkiHelsinkiFinland; ^3^ Department of Internal MedicineTampere University HospitalTampereFinland; ^4^ University of TampereTampereFinland; ^5^ Department of Internal MedicineTurku University HospitalTurkuFinland; ^6^ Department of Internal MedicineKuopio University HospitalKuopioFinland; ^7^ Department of Internal MedicineOulu University HospitalOuluFinland

**Keywords:** Acute lymphoblastic leukemia, asparaginase, cerebral venous thrombosis, risk factors

## Abstract

Cerebral venous thrombosis (CVT) covers up to a third of all venous thromboses (VTs) detected in patients with acute lymphoblastic leukemia (ALL). It usually hampers patients' lives and may also endanger efficient leukemia treatment. Although many factors have been suggested to account for an elevated risk of VTs in patients with ALL, there still is a lack of studies focusing on CVTs and especially in the setting of adult ALL patients. We studied in our retrospective population‐based cohort the occurrence, characteristics, as well as risk factors for VTs in 186 consecutively diagnosed Finnish adult ALL patients treated with a national pediatric‐inspired treatment protocol ALL2000. In the risk factor analyses for VTs we found a distinction of the characteristics of the patients acquiring CVT from those with other kinds of VTs or without thrombosis. In contrast to previous studies we were also able to compare the effects of asparaginase in relation to CVT occurrence. Notably, more than half of the CVTs were diagnosed prior the administration of asparaginase which accentuates the role of other risk factors on the pathophysiology of CVT compared to truncal or central venous line (CVL) VTs in adult ALL patients.

## Introduction

Venous thromboses cause high mortality in cancer patients 1, 2. Prior studies conducted mainly on pediatric acute lymphoblastic leukemia (ALL) patients report associations of venous thromboses (VTs) with patient‐ (high body mass index (BMI), advanced age, comorbidities), disease‐ (ALL subtype), and treatment‐related factors (asparaginase, steroids, and intrathecal chemotherapy) 3, 4, 5, 6, 7, 8, 9, 10. Most VTs including cerebral venous thromboses (CVTs) in ALL patients occur during the first 2 or 3 months of leukemia treatment 3, 4, 5.

CVTs cover up to over 30% of all cases of VTs in ALL patients. It leads to dramatic consequences such as epilepsy and cognitive or focal deficits in a significant amount of encountered patients 3, 6. Etiological factors identified in nonleukemia patient cohorts include female sex, hormonal manipulation (e.g., oral contraceptives), certain malignancies, and head trauma, but they explain less than half of the CVT cases 11, 12. Although CVT is a relatively common complication in ALL patients, we still do not know the specific biological basis of this event 3, 10.

CVTs may be hard to diagnose because the symptoms vary from mild headaches to life‐threatening seizures or nausea with concomitant intracranial hypertension. The usual time lag between the onset of symptoms and diagnosis is about 2 weeks 11, 12.

Asparaginase is considered efficient and essential in ALL treatment. It depletes free asparagine and glutamine from the extracellular fluid. This leads to apoptosis of the malignant lymphoblastic cells as their cell growth and division are highly dependent of these circulating amino acids 13. Aside from the aimed antileukemic properties, asparaginase exposes patients to multiple adverse effects such as VTs. Other suspected, although not unanimously identified, risk factors of VTs in ALL patients include high BMI, T‐cell ALL (T‐ALL), and the concomitant use of steroids 14, 15, 16, 17. As to our knowledge, reports on whether the risk factors for CVTs and truncal or central venous line (VCL) VTs in ALL patients are alike, still lack.

We performed a population‐based retrospective registry study on the Finnish ALL patients treated with a national ALL2000 study protocol during 2000–2012. We aimed to discover the potential distinction of the risk factors for CVTs and other VTs in ALL patients. Khorana score has been used for definition of patients in high risk of venous thrombosis in solid tumor malignancies 18. In this study we also aimed to retrospectively monitor, whether basic laboratory tests and morphological factors detectable at ALL diagnosis could reveal patients in a high risk of a CVT.

## Patients and methods

The study initially comprised of 201 consecutive adult ALL patients (aged 16–65 years) treated with a national protocol ALL2000 (results unpublished; MEA vs. CVAD as induction, CVAD as the first consolidation, and asparaginase introduced in the second consolidation; Table 1) in the five Finnish university hospitals (Helsinki, Kuopio, Oulu, Tampere, and Turku) and Vaasa Central Hospital during 1999–2012. Patients were followed up to April 2015 (median total follow‐up: 4.2 years, range: 0.02–14.9 years) after which the analysis was conducted. Patient consent was obtained according to the Declaration of Helsinki and the study was approved by the respective Ethics committee of each participating hospitals. Of 201 patients, 186 were included in our analysis and 15 excluded by following criteria: insufficient patient records (*n* = 11), false primary diagnose (one lymphoma and one chronic lymphoblastic leukemia), and prior acute leukemia (*n* = 2) (Fig. S1).

**Table 1:**
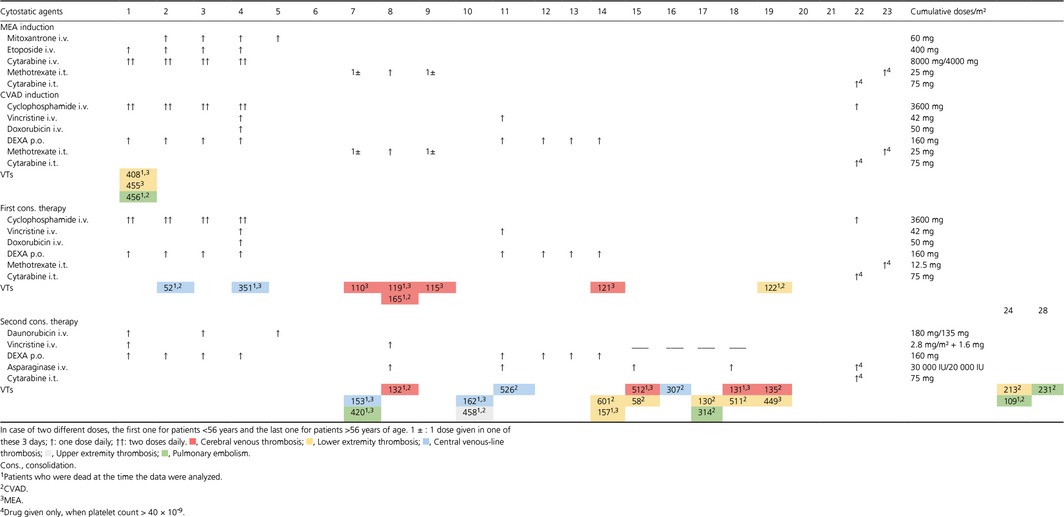
Occurrence of venous thromboses in the ALL2000 cytostatic treatment. This table shows the cytostatic protocol and the time of venous thrombosis (VT) diagnosis in the first three treatment blocks of the ALL2000 study. Intrathecal cytostatic and asparaginase treatments are highlighted with colors (light orange and light red, respectively). Patients with venous thromboses (VTs, numbers represent patient codes) are also highlighted with colors that are explained in the footnote

In the ALL2000 study patients were randomized into two different induction groups, CVAD and MEA, after which they received identical consolidation therapies (Table 1). CVAD was given to 96 (52%) and MEA to 90 (48%) of the patients. *Escherichia coli*‐derived asparaginase was introduced at 8th day of the second consolidation block. In our study the median interval from the ALL diagnosis to the first actualized dose of asparaginase was 64 days (range: 43–196 days). Follow‐up for the occurrence of VTs (median: 88 days, range: 7–219 days) was the time measured from leukemia diagnosis to the end of second consolidation block, allogeneic stem cell transplantation (alloSCT), or death. Primary endpoints of our study were a VT, alloSCT, or death. Only two of our study patients received prophylactic anticoagulation during leukemia treatment. Their indication for prophylaxis was atrial fibrillation.

The clinical phenotypes and basic laboratory values of the patients were recorded and included in our analysis. Detailed description of these parameters is shown in Table 2 and Table S1. All data were collected from patient charts. Only VTs confirmed by radiological imaging techniques (ultrasound, computed tomography, or magnetic resonance imaging) were included in the analysis. Patient characteristic at the time of ALL diagnosis (Table 2), clinical characteristics of the VT patients (Table 3), and the data used in the analysis (Table S1) are shown.

**Table 2:** Patient characteristics at ALL diagnosis. Patients were divided according to ALL disease subtypes

Patients	All (*n* = 186)	pre‐B (*n* = 118)	Ph+ (*n* = 37)	T (*n* = 31)
Variables	Yes, *n* (%)/Median (range)	Yes, *n* (%)/Median (range)	Yes, *n* (%)/Median (range)	Yes, *n* (%)/Median (range)
Gender (Male)	111 (60)	71 (60)	20 (54)	21 (65)
Age	40.9 (16.1‐65.8)	39.9 (16.1‐65.8)	48.9 (19.2‐64.6)	35.6 (17.0‐61.5)
BMI	25.5 (17.0‐46.5)	25.5 (17.5‐46.5)	25.7 (19.7‐40.3)	25.0 (17.0‐39.5)
Extramedullary leukemia	44 (24)	21 (18)	3 (9)	19 (61)
Abdomen, mediastinum, skin, and/or lymph nodes	39 (21)	18 (16)	2 (5)	19 (61)
CNS leukemia	4 (3)	3 (2)	1 (3)	0 (0)
Prior comorbidities				
No comorbidities	118 (64)	70 (60)	24 (65)	24 (77)
At least one comorbidity	66 (36)	46 (40)	13 (35)	7 (23)
Hypertension	18 (10)	15 (13)	2 (5)	1 (3)
Hypercholesterolemia	12 (6)	10 (8)	2 (5)	0 (0)
Diabetes	10 (5)	7 (6)	2 (5)	1 (3)
Asthma	7 (4)	6 (5)	0 (0)	1 (3)
Thyroid disorder	7 (4)	4 (3)	3 (8)	0 (0)
Atrial fibrillation	2 (1)	0 (0)	2 (5)	0 (0)
Myocardial infarction	3 (2)	3 (3)	0 (0)	0 (0)
Epilepsy	3 (2)	2 (2)	0 (0)	1 (3)
Ulcerative colitis	4 (2)	4 (3)	0 (0)	0 (0)
Previous cancer	7 (4)	4 (3)	3 (8)	0 (0)
Prior venous thrombosis	2 (1)	2 (2)	0 (0)	0 (0)
Other factors				
Smoking (current/prior)	32 (17)	18 (15)	6 (16)	8 (26)
Infection less than a week prior to ALL diagnosis	21 (12)	12 (10)	4 (12)	5 (16)
BMI > 30	31 (17)	23 (21)	4 (11)	4 (13)
Hormonal therapy (progestin/estrogen) at diagnosis	6 (3)	3 (3)	1 (3)	2 (6)
Anticoagulation at diagnosis	3 (2)	2 (2)	1 (3)	0 (0)

Philadelphia‐positive (Ph+) patients were statistically significantly older than precursor B‐ALL (pre‐B) and T‐ALL (T) patients (*P* = 0.032 and *P* = 0.009, respectively). T‐ALL patients had significantly more extramedullary leukemia compared to other disease subtypes (*χ*2: 31.984, *P* = 0.001). No further statistically significant differences between ALL subtypes were detected in other patient characteristics.

BMI, body mass index; CNS, Central nervous system.

**Table 3:** Clinical characteristics of patients with venous thromboses

Type of event variables	No venous thrombosis (*n* = 155)	All venous thromboses (*n* = 31)	Other venous thromboses (*n* = 22)	CVTs (*n* = 9)	All vs. no venous thromboses	Other vs. no venous thromboses	CVTs vs. no venous thromboses	CVTs vs. other venous thromboses
Prechemotherapy laboratory values	Median (range)	Median (range)	Median (range)	Median (range)	*P*‐value	*P*‐value	*P*‐value	*P*‐value
Hemoglobin	94.5 (35–172)	106 (39–159)	107 (68–159)	105 (39–136)	0.163	0.265	0.307	0.695
Platelets	56.0 (4–445)	56.0 (3–291)	57.5 (7–291)	35.0 (3–204)	0.869	0.697	0.309	0.306
Leukocytes	11.0 (0.6–307)	17.9 (1.3–237)	16.3 (1.3–237)	18.9 (2–221)	0.333	0.642	0.269	0.500
CRP	18.5 (1–419))	17.0 (2–221)	12.0 (2–139)	43.0 (2–221)	0.863	0.236	0.123	0.033
D‐Dimer	1.80 (0.02–97)	2.80 (0.1–71)	2.40 (0.2–12)	5.40 (0.1–71)	0.539	0.899	0.318	0.236
B‐blasts	4.70 (0–260)	11.6 (0–219)	8.30 (0–219)	13.7 (0–217)	0.431	0.746	0.300	0.497
Patient and disease characteristics								
Age	41.2 (16.1–56.4)	40.6 (18.2–65.3)	43.6 (18.2–65.3)	27.1 (18.6–58.2)	0.852	0.275	0.166	0.056
BMI	25.5 (17.5–46.5)	25.3 (17.0–39.5)	27.0 (20.2–39.5)	21.5 (17.0–26.0)	0.673	0.038	0.018	0.002
Gender, Male, *n* (%)	95 (61)	17 (55)	13 (62)	4 (44)	0.547	0.907	0.335	0.457
Disease subgroups	Yes, *n* (%)	Yes, *n* (%)	Yes, *n* (%)	Yes, *n* (%)				
precursor B‐cell ALL	98 (63)	20 (65)	18 (82)	2 (22)				
Philadelphia‐positive B‐cell ALL	34 (22)	3 (14)	1 (5)	2 (22)				
T‐cell ALL	23 (15)	8 (38)	3 (14)	5 (56)	0.148	0.148	0.005	0.007
Extramedullary leukemia	30 (19)	9 (30)	4 (18)	5 (56)	0.227	0.942	0.010	0.037
CNS leukemia	4 (3)	0 (0)	0 (0)	0 (0)	0.366	0.444	0.626	
≥ 1 Comorbidities	51 (33)	15 (48)	12 (55)	3 (33)	0.111	0.053	1.000	0.283
Smoking history	24 (16)	8 (26)	6 (27)	3 (33)	0.080	0.183	0.171	0.210
Infection at diagnosis	16 (11)	5 (17)	3 (14)	2 (29)	0.327	0.695	0.152	0.362
Treatment‐associated factors at venous thrombosis								
Day of the thrombosis			68 (0–217)	43 (34–126)				0.349
Central venous‐line catheter at thrombosis			8 (44)	4 (44)				1.000
Asparaginase before thrombosis			15 (68)	4 (44)				0.218
Intrathecal chemotherapy a week before thrombosis			3 (14)	0 (0)				0.244
Dexamethasone a week before thrombosis			21 (95)	9 (100)				0.516

BMI, Body mass index; CVT, Central venous thrombosis.

### Statistical analyses

SAS statistical software 9.3 (SAS Institute, Care, NC) and IBM SPSS statistics 22 were used for the statistical analysis. Mann–Whitney U or Pearson Chi‐squared (*χ* ^2^) tests were used in univariate analysis for patient characteristics depending on the data characteristics. Cox regression model was plotted with time‐dependent covariate (asparaginase treatment) and the risk score model for CVT by using FREQ procedure. All analyses were two sided and *P* < 0.05 was considered statistically significant.

## Results

In total, 31 (17%) patients suffered from a VT, of whom nine (27%) had a CVT (Table 3). Other types of VTs (*n* = 22) included ten lower and one upper extremity deep venous thromboses, six central venous‐line thromboses, and five pulmonary embolisms. The exact dates of all VTs are shown on Table 1. CVTs occurred mostly at the first consolidation therapy which was earlier than other recorded VTs (median: 43 days, range: 34–126 and median: 68 days, range: 1–127 and, respectively; *P* > 0.05).

A detailed description of the patient characteristics and distribution of them among leukemia or thrombosis subtypes is given in Table 3. Distribution of CVAD or MEA induction among patients suffering from thrombosis during the follow‐up is shown in Table 1.

The univariate analysis showed a significantly lower median BMI (21.5 kg/m^2^, range: 17.0–26.0 kg/m^2^) of CVT patients compared to patients with other VTs (*P* = 0.002) or without VTs (*P* = 0.018), respectively. Furthermore, CVT patients had more T‐ALL (5/9, 56%) and extramedullary leukemia (5/9, 56%) than patients with other VTs (3/22, (14%), *P* = 0.007 and 4/22, (18%), *P* = 0.037) and no VTs (23/155, (15%), *P* = 0.005 and 30/155, (19%), *P* = 0.010) (Table 3). CVT patients had a significantly higher CRP compared to patients with other VTs (*P* = 0.033), but a difference in leukocyte and platelet counts did not reach statistical significance (Table 3). Detailed characteristics of patients with CVTs are shown in Table 4. Most of the CVTs (5/9 (56%)) occurred before the first administration of asparaginase and we did not detect association of asparaginase with CVT in the Cox regression model (*χ*2: 0.000, *P* = 0.995). Lower BMI and T‐ALL subtype remained significant factors in also this analysis (*χ*2: 3.926, *P* = 0.048 and *χ*2: 4.8708, *P* = 0.025, respectively). Of the CVT patients, 8/9 (89%) had been on oral steroids (dexamethasone), but none had received intrathecal chemotherapy (cytarabine or methotrexate) a week prior the thrombosis.

**Table 4:** Characteristic of patients with cerebral venous thromboses

Patient characteristics and laboratory values at diagnosis													Characteristics of CVTs			Treatment‐related factors		
Nb	Sex	Day^	Disease subtype	EM disease	CNS leukemia	Age	BMI	Plts	Hb	Leuk	CRP	D‐dimer	Symptoms	Site	Imaging	ASP	DEXA^^	It. therapy^^
1	F	34	Ph+	No	No	41.0	23.6	3	98	9.8	186	71	Right hemiparesis, vertigo, speech deficit^a^	SSS, left transverse sinus	MRI	Never	Yes	No
2	F	39	pre‐B	No	No	20.8	21.5	27	39	18.9	4	0.1	Headache^a^	SSS	CT + MRI	Never	Yes	No
3	M	43	T	Yes	No	20.7	19.6	204	125	2	10	0.2	Left hemiparesis, seizures, headache^a^	SSS	MRI	Never	Yes	No
4	F	43	pre‐B	Yes	No	26.3	20.4	72	105	221	73	5.9	Numbness of the left arm, face, and tongue^a^	Cortical veins	MRI	′Never	Yes	No
5	M	73	T	Yes	No	37.0	23.7	22	85	35.7	23	35	Left hemiparesis and numbness of the left side^a^	SSS	MRI	Yes	Yes	No
6	M	50	T	Yes	No	27.1	21.0	78	120	3.8	131	5.9	Headache^a^	SSS, venous infarctions	MRI	Yes	Yes	No
7	F	65	T	No	No	18.5	17.0	74	113	188	33	5.4	Numbness and paresis of the left arm^b^	SSS	CT + MRI	Yes	Yes	No
8	F	42	Ph+	No	No	48.2	26.0	35	136	64.4	221	0.5	Right hemiparesis, speech deficit^b^	SSS, cortical veins, hemorrhage	CT + MRI	Never	No	No
9	M	126	T	Yes	No	40.2	25.4	30	104	15	43	3.6	Headache, fatigue	SSS, sinus rectus	MRI	Yes	Yes	No

Nb, patient number; Day^, number of days between ALL diagnosis and the CVT; EM, extramedullary; CNS, central nervous system; BMI, body mass index; Plt, platelet count; Hb, hemoglobin; Leuk, leukocytes; ASP, asparaginase; DEXA, dexamethasone; I.t., intrathecal; ^^, less than a week before the CVT. Parameters used in the table: F, female; M, male; Ph+, Philadelphia‐positive ALL; pre‐B, precursor B‐ALL; T, T‐ALL.

a Prodromal symptoms

b Prodromal syndromes that occurred before the introduction of asparaginase; SSS: superior sagittal sinus; MRI: magnetic resonance imaging; and CT: computer tomography.

Patients with truncal or CVL VTs expressed typical general risk characteristics for VTs in the univariate analysis. They were older, had more comorbidities, and had a significantly higher median BMI (27.1 kg/m^2^, range: 20.2–39.5, *P* = 0.038) compared to patients without VTs. (Table 3). Only 5/22 (32%) of the patients suffered from thrombosis before the first dosing of asparaginase and the Cox regression analysis showed a clear association of asparaginase treatment with occurrence of truncal or CVL VTs (*χ* ^2^: 6.850, *P* = 0.0089). Furthermore, 21/22 (95%) received oral steroids and 3/22 (14%) intrathecal treatment less than a week prior to the VT.

Extramedullary leukemia was found in 44 of 186 (24%) patients, and distribution of extramedullary leukemia is shown in Table S2. Of the nine CVT patients, two had a mediastinal tumor mass, two markedly enlarged lymph nodes, and one leukemic skin infiltration at diagnosis. Ninety‐eight (53%) of all patients, 15 (48%) of patients with other VTs, and 5 (56%) of patients with a CVT were alive at the time the data were analyzed (Table S3). Most of all VTs (in total 52%) were diagnosed and treated in Helsinki University Hospital (Table S4). No statistically significant differences between the incidence rates of VTs were detected between the hospitals.

**Figure 1:**
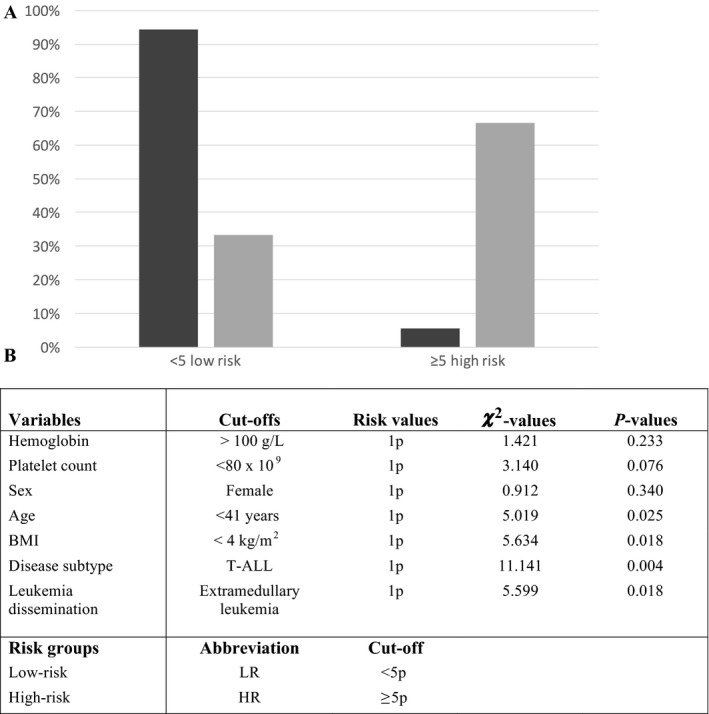
Risk score model for cerebral venous thrombosis. (A) Shows graphics of the risk score model for cerebral venous thrombosis (CTV) where CVT patients (light gray bars, *n* = 9) are compared to patients without CVT (dark gray bars, *n* = 177) at the time of ALL diagnosis. Percentages of amount of patients belonging to each risk group (x‐axis) are shown on the y‐axis. Of CVT and no CVT patients, 6/9 (67%) and 10/177 (6%) scored ≥ 5 points (high‐risk group), respectively. (B) Shows variables and cut‐offs used in the risk score model for CVT. Cut‐offs with *P*‐value < 0.05 were used. Hemoglobin cut‐off was based on Khorana risk score model 15 and female sex based on the female dominance in the CVT cases 9.

We also constructed a preliminary CVT risk score model based on our results, prior knowledge of CVT risk factors, and Khorana risk score model 11, 12, 18. Age, gender, BMI, disease subtype, leukemia dissemination, hemoglobin, and platelet counts at ALL diagnosis were included in the score. Ranking limits (0 or 1 point/parameter) were selected according to the association study results previously published 18. A risk score of ≥5 points equals to a high risk for CVT (Fig. 1). Patients in the high‐risk group represented more than 20‐fold risk for CVT compared to the patients in the low‐risk group (hazard ratio = 20.841, *P* < 0.0001, 95% CI: 5.208–83.401). The score model showed an excellent specificity and a moderate sensitivity (NPV: 0.982, PPV: 0.375).

## Discussion

Venous thromboses often compromise the effective treatment of ALL. We performed a detailed analysis of the occurrence and risk factors for VTs in a consecutive series of adult patients diagnosed in Finland in 2000–2012 and treated according to a national study protocol ALL2000. The incidence rates of VTs were comparable to previous reports 10, 12, 15, 16, 17. Risk factors for truncal or CVL VTs were as hypothesized and reflected many of the traditional hazards for thrombosis detected in the general population. These factors, however, were distinct from those detected in patients with CVT in our study cohort.

Namely, patients acquiring CVT were younger and had a statistically significantly lower BMI and higher incidences of extramedullary and T‐cell leukemia compared to patients with no VTs and truncal or CVL VTs. A study made by Zuurbier et al. showed no differences in age (median 33 vs. 33 years) between CVT and no CVT patients, whereas CVT patients in a study conducted by Couturier et al. showed age distribution (median 29 vs. 33 years) near to our results but without a statistical significance 14, 15. In comparison to our results these discrepancies may be explained by a different age distribution of the patient population (16–65 and 16–18 to 59 years, respectively). Young age as risk factor for CVT is supported by the review by McBane et al., where median of the patients receiving CVT were at the age of early forties 11. The distinctively low BMI in CVT patients in our analysis is likely explained by the young age of the affected patients.

CVT is a severe complication that may lead to difficult sequelae 11, 12, 14. It often causes delays or truncations in the treatment of ALL. Hence, we think that clinicians should be aware that not only the overweighed and older patients are at risk of a serious thrombotic complications. As delays in diagnoses of CVTs are frequent our results may also carry on impact in sensitizing clinicians to order proper radiological investigations for patients with neurological symptoms.

Mediastinal mass may increase the risk of thrombosis by, for example, increasing mediastinal pressure that could impair venous flow from the cerebral veins 6. Two (22%) of the nine CVT patients (Table 4) had a mediastinal mass at ALL diagnosis in our study. Due to the limited number of the study patients, the etiological role of these masses in CVT occurrence cannot be estimated. Compared to the ALL‐subtype distribution of all ALL2000 patients (Table 2 and Table 3) our CVT patients had 3.3‐fold incidence of T‐ALL. This is more than reported previously (2‐ and 1.5‐fold) 14, 15. T‐ALL is more frequent in males which likely explains the even distribution of our CVT cases between genders in our analysis. T‐ALL is also recurrently associated with an inferior outcome of ALL 19. These factors—age, BMI, extramedullary leukemia, and T‐ALL—together with the prechemotherapy trend of low platelet and high leukocyte counts might reflect an aggressive form of leukemia that potentially and specifically predisposes patients for CVTs. Unfortunately, neither detailed genomic data on inherited factors affecting patients' potential thrombophilia were available nor biobank samples collected due to technical limitations in the molecular genetic diagnostics during the era of ALL2000 data collection.

Contrary to current consent, over half (56%) of the patients suffered from CVT before the introduction of asparaginase in our study cohort 10. In addition, two of the nine CVT patients suffered from prodromal symptoms before the first dose of asparaginase (problems with gait *n* = 1, persistent headaches *n* = 1) (Table 3). This could have reflected an already ongoing thrombotic process. Unlike ALL2000 protocol, most current ALL treatment protocols introduce asparaginase at induction. These types of study settings might therefore partly overweight the causal role of asparaginase for CVTs. Our results demonstrate for the first time a minor associative role of asparaginase treatment on the occurrence of CVT. The analysis is unique because it enabled us to study the risk factors of an early occurring CVT in adult ALL patients without the effect of asparaginase during the first two treatment blocks where the risk of CVT is at the highest. We suggest that this novel finding further strengthens the idea of the differences in pathophysiology of CVT, and truncal or CVL‐related thrombosis. We also suggest that ALL disease itself might play a bigger role in the development of CVT than thought before.

Central nervous system (CNS) malignancies and intrathecal chemotherapy may contribute to the risk of CVT development, by, for example, increasing the brain tissue prothrombin levels, or by damaging the blood–brain barrier 11, 14. None of our patients had received intrathecal chemotherapy shortly before the thrombosis (Table 3). Although none of our CVT patients had a detectable CNS leukemia in the morphological analysis of the cerebrospinal fluid, it is still possible that a minimal, occult CNS leukemia could account for an increased risk of CVT. Regrettably, antithrombin levels that have been shown to increase the risk of thrombosis were not routinely collected at the time of patient enrolment in this study 9. A recent study on acute myeloid leukemia patients suggests that a situation parallel with disseminated intravascular coagulopathy (i.e., low platelet count, low TT%, and high D‐dimer) could increase the risk of venous thrombosis 20. Unfortunately, although our CVT patients showed a decreased level of platelets and elevated level of D‐dimer, no TT% was measured at the time of ALL diagnosis. This idea could not therefore be evaluated in our cohort.

Based on Khorana risk score model, results obtained in our study, and the knowledge of common CVT risk factors, we built a preliminary risk score model for CVT 11, 12, 18. Retrospective study design and limited number of patients, however, restrict our analysis and it therefore needs to be validated in a larger and prospective study cohort.

Our study is probably the first one to analyze differences in potential risk factors of a CVT compared with patient groups of both no VTs and with other VTs in adult ALL. Surprisingly, asparaginase treatment did not show association with the occurrence of CVTs in our study setting. Patients with a CVT were also leaner and younger than other subjects. We suggest that rather than imputed acquired risk factors for VTs, such as asparaginase, the characteristics of the ALL disease itself might play a significant role in the development of CVT in adult ALL patients. We think that more studies aiming at validation of an individualized CVT risk estimation in ALL patients are needed for. Genetic factors related both to leukemia and inherited thrombophilia should also be investigated and potentially taken into account.

## Conflict of Interest

None declared.

## Supporting information


**Figure S1.** Patients enrolled at the study. ALL: acute lymphoblastic leukemia; CCL: chronic lymphoblastic leukemia.

**Table S1.** Data used in the ALL analysis.

**Table S2.** Distribution of extramedullary leukemia among ALL patients. Sites and the incidence rate of extramedullary leukemia are shown on the table. In case of several extramedullary sites, central nervous system (CNS‐) leukemia, mediastinal mass, spleen and lymph nodes were considered as the major site of an extramedullary leukemia. The column proportions do not differ significantly from each other at the 0.05 level. VT: venous thrombosis, CVT cerebral venous thrombosis, CVL: central venous line.

**Table S3.** Survival of patients in different groups. The column proportions do not differ significantly from each other at the 0.05 level. VT: venous thrombosis, CVT cerebral venous thrombosis, CVL: central venous line.

**Table S4.** Distribution of venous thromboses in different hospitals. Each subscript letter denotes a subset of Hospital categories whose column proportions do not differ significantly from each other at the 0.05 level. VT: venous thrombosis, CVT cerebral venous thrombosis, CVL, central venous line.
