# Supplementary figures and images for: A minor role of asparaginase in predisposing to cerebral venous thromboses in adult acute lymphoblastic leukemia patients
Source: Cancer Med. 2017 May 15;6(6):1275–85. doi: 10.1002/cam4.1094 (PMC5463063; doi:10.1002/cam4.1094)

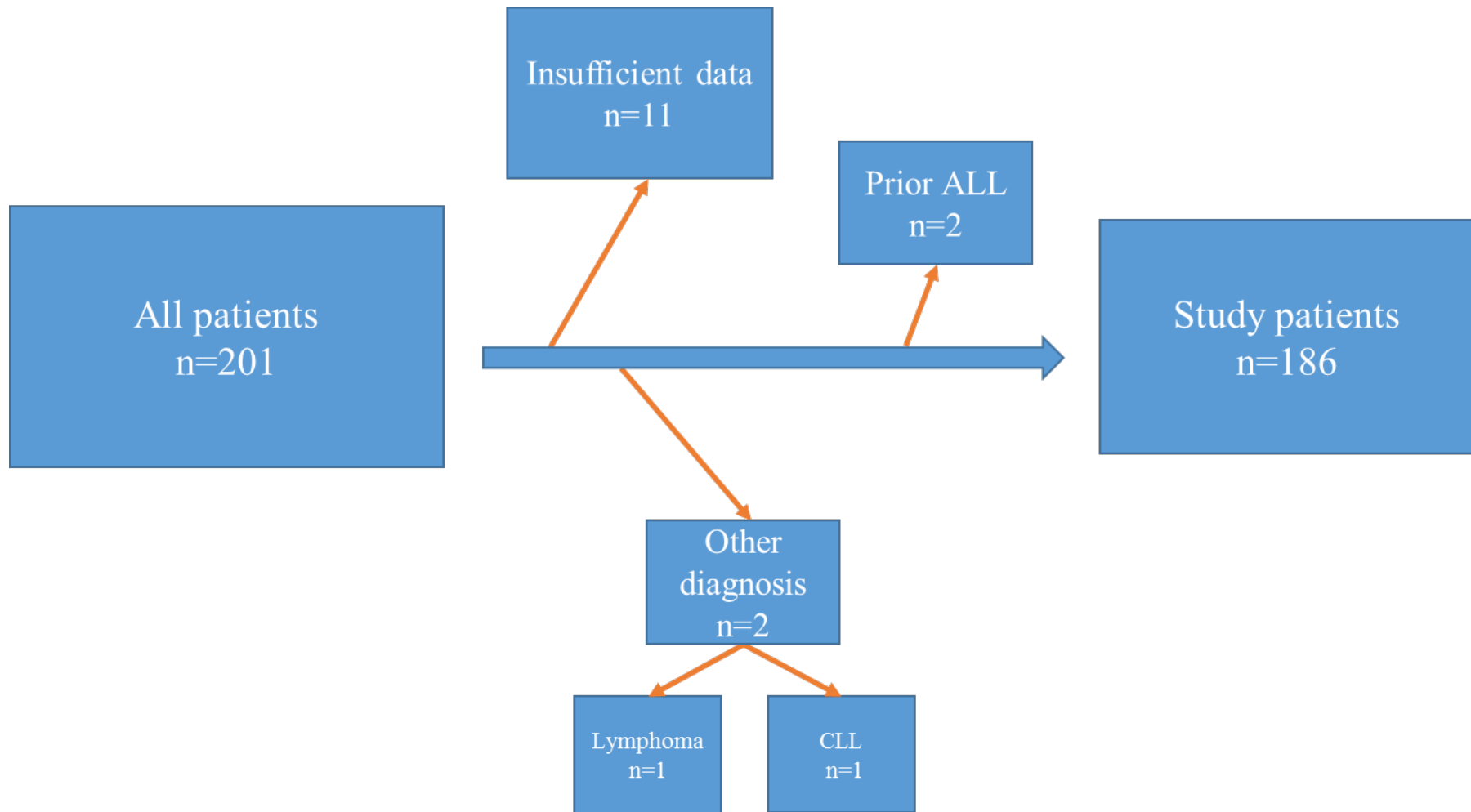

Supplement: Supplementary file 1 — Figure S1. Patients enrolled at the study. ALL: acute lymphoblastic leukemia; CCL: chronic lymphoblastic leukemia. [file CAM4-6-1275-s001.pdf]
