# Supplementary material for: A minor role of asparaginase in predisposing to cerebral venous thromboses in adult acute lymphoblastic leukemia patients
Source: Cancer Med. 2017 May 15;6(6):1275–85. doi: 10.1002/cam4.1094 (PMC5463063; doi:10.1002/cam4.1094)
Supplement: Supplementary file 2 — Table S1. Data used in the ALL analysis. [file CAM4-6-1275-s002.docx]

| Variables | No. Of all patients (% of all cases) | No. of patients with no venous thrombosis (% of all cases) | No. of patients with other venous thromboses (% of all cases) | | No. of CVT patients (% of all cases) |
| --- | --- | --- | --- | --- | --- |
| **Data collected at diagnosis** |  |  |  |  | |
| Sex | 186 (100%) | 155 (100%) | 22 (100%) | 9 (100%) | |
| Age | 186 (100%) | 155 (100%) | 22 (100%) | 9 (100%) | |
| Induction group | 186 (100%) | 155 (100%) | 22 (100%) | 9 (100%) | |
| Disease group | 186 (100%) | 155 (100%) | 22 (100%) | 9 (100%) | |
| Extramedullary leukemia | 184 (98,9%) | 154 (99.4%) | 21 (95.5%) | 9 (100%) | |
| CNS-leukemia | 183 (98.4%) | 153 (98.7%) | 21 (95.5%) | 9 (100%) | |
| BMI | 180 (96.8%) | 151 (97.4%) | 22 (100%) | 9 (100%) | |
| Hemoglobin | 186 (100%) | 155 (100%) | 22 (100%) | 9 (100%) | |
| Platelet count | 186 (100%) | 155 (100%) | 22 (100%) | 9 (100%) | |
| Leukocyte count | 186 (100%) | 155 (100%) | 22 (100%) | 9 (100%) | |
| D-dimer | 157 (84.4%) | 130 (83.9%) | 18 (81.8%) | 9 (100%) | |
| CRP | 182 (97.8%) | 152 (98.1%) | 21 (95.5%) | 9 (100%) | |
| Blood blast count | 185 (99.5%) | 155 (100%) | 21 (95.5%) | 9 (100%) | |
| Comorbidities* at ALL diagnosis | 182 (97.8%) | 151 (97.4%) | 22 (100%) | 9 (100%) | |
| Prior cancer | 183 (98.4%) | 152 (98.1%) | 22 (100%) | 9 (100%) | |
| Smoking (current/prior) | 183 (98.4%) | 152 (98.1%) | 22 (100%) | 9 (100%) | |
| Hormonal therapy (progestin/estrogen) at diagnosis | 180 (96.8%) | 149 (96.1%) | 22 (100%) | 9 (100%) | |
| Anticoagulation treatment at diagnosis | 184 (98,9%) | 153 (98.7%) | 21 (95.5%) | 9 (100%) | |
| Infection less than a week prior to ALL diagnosis | 177 (95.2%) | 148 (95.5%) | 22 (100%) | 9 (100%) | |
| **Data collected during treatment** |  |  |  |  | |
| Central venous-line catheter | 144 (77.4%) | 117 (75.5%) | 18 (81.8%) | 9 (100%) | |
| Date of asparaginase introduction | 175 (94.1%) | 144 (92.9%) | 22 (100%) | 9 (100%) | |
| Date of the latest dexamethasone therapy prior to thrombosis |  |  | 22 (100%) | 9 (100%) | |
| Date of the latest intrathecal chemotherapy prior to thrombosis |  |  | 22 (100%) | 9 (100%) | |
| Platelet transfusion less than a week prior to thrombosis |  |  | 19 (86%) | 8 (89%) | |
| RBC transfusion less than a week prior to thrombosis |  |  | 20 (91%) | 8 (89%) | |
| Follow-up | 186 (100%) | 155 (100%) | 22 (100%) | 9 (100%) | |
