# Supplementary material for: A minor role of asparaginase in predisposing to cerebral venous thromboses in adult acute lymphoblastic leukemia patients
Source: Cancer Med. 2017 May 15;6(6):1275–85. doi: 10.1002/cam4.1094 (PMC5463063; doi:10.1002/cam4.1094)
Supplement: Supplementary file 3 — Table S2. Distribution of extramedullary leukemia among ALL patients. Sites and the incidence rate of extramedullary leukemia are shown on the table. In case of several extramedullary sites, central nervous system (CNS‐) leukemia, mediastinal mass, spleen and lymph nodes were considered as the major site of an extramedullary leukemia. The column proportions do not differ significantly from each other at the 0.05 level. VT: venous thrombosis, CVT cerebral venous thrombosis, CVL: central venous line. [file CAM4-6-1275-s003.pdf]

Supplementary table II. Distribution of extramedullary leukemia among ALL patients. Sites and the incidence rate of extramedullary leukemia is shown on the table. In case of several extramedullary sites, central nervous system (CNS-) leukemia, mediastinal mass, spleen and lymph nodes were considered as the major site of an extramedullary leukemia. The column proportions do not differ significantly from each other at the 0.05 level.

Type of extramedullary disease	VT groups	No thrombosis	Lower extremity VT	Upper extremity VT	CVT	Pulmonary embolism	CVL thrombosis	Total
No extramedullary leukemia	Count	119	9	1	4	5	3	142
	% within VT groups	76,8 %	90,0 %	100,0 %	44,4 %	100,0 %	50,0 %	76,3 %
Mediastinum	Count	7	0	0	2	0	1	10
	% within VT groups	4,5 %	0,0 %	0,0 %	22,2 %	0,0 %	16,7 %	5,4 %
Lymph nodes	Count	9	0	0	2	0	1	11
	% within VT groups	5,8 %	0,0 %	0,0 %	22,2 %	0,0 %	16,7 %	5,9 %
Spleen	Count	14	1	0	0	0	1	16
	% within VT groups	9,0 %	10,0 %	0,0 %	0,0 %	0,0 %	16,7 %	8,6 %
Skin	Count	1	0	0	1	0	0	2
	% within VT groups	0,6 %	0,0 %	0,0 %	11,1 %	0,0 %	0,0 %	1,1 %
Liver	Count	1	0	0	0	0	0	1
	% within VT groups	0,6 %	0,0 %	0,0 %	0,0 %	0,0 %	0,0 %	0,5 %
CNS leukemia	Count	4	0	0	0	0	0	4
	% within VT groups	2,6 %	0,0 %	0,0 %	0,0 %	0,0 %	0,0 %	2,2 %
Total	Count	155	10	1	9	5	6	186
	% within VT groups	100,0 %	100,0 %	100,0 %	100,0 %	100,0 %	100,0 %	100,0 %

VT: venous thrombosis, CVT cerebral venous thrombosis, CVL: central venous-line.
