# Supplementary material for: A minor role of asparaginase in predisposing to cerebral venous thromboses in adult acute lymphoblastic leukemia patients
Source: Cancer Med. 2017 May 15;6(6):1275–85. doi: 10.1002/cam4.1094 (PMC5463063; doi:10.1002/cam4.1094)
Supplement: Supplementary file 4 — Table S3. Survival of patients in different groups. The column proportions do not differ significantly from each other at the 0.05 level. VT: venous thrombosis, CVT cerebral venous thrombosis, CVL: central venous line. [file CAM4-6-1275-s004.docx]

| Status |  | No thrombosis | Lower extremity VT | Upper extremity VT | CVT | Pulmonary embolism | CVL thrombosis | Total | |
| --- | --- | --- | --- | --- | --- | --- | --- | --- | --- |
| Alive | Count | 83 | 7 | 0 | 4 | 2 | 2 | | 98 |
|  | % within patient groups | 53.5% | 70.0% | 0.0% | 44.4% | 40.0% | 33.3% | | 52.7% |
| Exitus | Count | 72 | 3 | 1 | 5 | 3 | 4 | | 88 |
|  | % within patient groups | 46.5% | 30.0% | 100.0% | 55.6% | 60.0% | 66.7% | | 47.3% |
| Total | Count | 155 | 10 | 1 | 9 | 5 | 6 | | 186 |
|  | % within patient groups | 100.0% | 100.0% | 100.0% | 100.0% | 100.0% | 100.0% | | 100.0% |
