# Supplementary material for: A minor role of asparaginase in predisposing to cerebral venous thromboses in adult acute lymphoblastic leukemia patients
Source: Cancer Med. 2017 May 15;6(6):1275–85. doi: 10.1002/cam4.1094 (PMC5463063; doi:10.1002/cam4.1094)
Supplement: Supplementary file 5 — Table S4. Distribution of venous thromboses in different hospitals. Each subscript letter denotes a subset of Hospital categories whose column proportions do not differ significantly from each other at the 0.05 level. VT: venous thrombosis, CVT cerebral venous thrombosis, CVL, central venous line. [file CAM4-6-1275-s005.docx]

|  |  |  |  |  |  |  |  |  |
| --- | --- | --- | --- | --- | --- | --- | --- | --- |
| VT groups | Hospitals | Helsinki | Kuopio | Oulu | Tampere | Turku | Vaasa | Total |
| Lower extremity VT | Count | 4 | 1 | 0 | 3 | 1 | 1 | 10 |
|  | % within Hospitals | 25,0 % | 50,0 % | 0,0 % | 50,0 % | 33,3 % | 100,0 % | 32,3 % |
| Lower extremity VT | Count | 0 | 0 | 0 | 1 | 0 | 0 | 1 |
|  | % within Hospitals | 0,0 % | 0,0 % | 0,0 % | 16,7 % | 0,0 % | 0,0 % | 3,2 % |
| CVT | Count | 8 | 0 | 0 | 0 | 1 | 0 | 9 |
|  | % within Hospitals | 50,0 % | 0,0 % | 0,0 % | 0,0 % | 33,3 % | 0,0 % | 29,0 % |
| Pulmonary embolism | Count | 1 | 1 | 1 | 2 | 0 | 0 | 5 |
|  | % within Hospitals | 6,3 % | 50,0 % | 33,3 % | 33,3 % | 0,0 % | 0,0 % | 16,1 % |
| CVL-thrombosis | Count | 3 | 0 | 2 | 0 | 1 | 0 | 6 |
|  | % within Hospitals | 18,8 % | 0,0 % | 66,7 % | 0,0 % | 33,3 % | 0,0 % | 19,4 % |
| Total | Count | 16 | 2 | 3 | 6 | 3 | 1 | 31 |
|  | % within Hospitals | 100,0 % | 100,0 % | 100,0 % | 100,0 % | 100,0 % | 100,0 % | 100,0 % |
